# Comparative Phylogenetic Studies on *Schistosoma japonicum* and Its Snail Intermediate Host *Oncomelania hupensis*: Origins, Dispersal and Coevolution

**DOI:** 10.1371/journal.pntd.0003935

**Published:** 2015-07-31

**Authors:** Stephen W. Attwood, Motomu Ibaraki, Yasuhide Saitoh, Naoko Nihei, Daniel A. Janies

**Affiliations:** 1 State Key Laboratory of Biotherapy, West China Hospital, West China Medical School, Sichuan University, Chengdu, People's Republic of China; 2 Department of Life Sciences, The Natural History Museum, London, United Kingdom; 3 School of Earth Sciences, The Ohio State University, Columbus, Ohio, United States of America; 4 Department of Environmental Parasitology, Tokyo Medical and Dental University, Tokyo, Japan; 5 Laboratory of Parasitology, School of Veterinary Medicine, Azabu University, Sagamihara, Japan; 6 Department of Medical Entomology, National Institute of Infectious Diseases, Tokyo, Japan; 7 Bioinformatics and Genomics, University of North Carolina at Charlotte, Charlotte, North Carolina, United States of America; Imperial College London, UNITED KINGDOM

## Abstract

**Background:**

*Schistosoma japonicum* causes major public health problems in China and the Philippines; this parasite, which is transmitted by freshwater snails of the species *Oncomelania hupensis*, causes the disease intestinal schistosomiasis in humans and cattle. Researchers working on *Schistosoma* in Africa have described the relationship between the parasites and their snail intermediate hosts as coevolved or even as an evolutionary arms race. In the present study this hypothesis of coevolution is evaluated for *S*. *japonicum* and *O*. *hupensis*. The origins and radiation of the snails and the parasite across China, and the taxonomic validity of the sub-species of *O. hupensis*, are also assessed.

**Methodology/Principal Findings:**

The findings provide no evidence for coevolution between *S*. *japonicum* and *O*. *hupensis*, and the phylogeographical analysis suggests a heterochronous radiation of the parasites and snails in response to different palaeogeographical and climatic triggers. The results are consistent with a hypothesis of East to West colonisation of China by *Oncomelania* with a re-invasion of Japan by *O*. *hupensis* from China. The Taiwan population of *S*. *japonicum* appears to be recently established in comparison with mainland Chinese populations.

**Conclusions/Significance:**

The snail and parasite populations of the western mountain region of China (Yunnan and Sichuan) appear to have been isolated from Southeast Asian populations since the Pleistocene; this has implications for road and rail links being constructed in the region, which will breach biogeographical barriers between China and Southeast Asia. The results also have implications for the spread of *S*. *japonicum*. In the absence of coevolution, the parasite may more readily colonise new snail populations to which it is not locally adapted, or even new intermediate host species; this can facilitate its dispersal into new areas. Additional work is required to assess further the risk of spread of *S*. *japonicum*.

## Author Summary

Schistosomiasis is a disease caused by a parasitic worm transmitted to humans by certain species of freshwater snails. In spite of several decades of intensive coordinated control schistosomiasis still infects around 1 million people in China. In order to understand the potential for spread of the disease into new areas and new snail species, it is helpful to know if the snails and parasites in China are coevolved; this means that evolutionary divergence in one group (the snails) is matched by a corresponding divergence in the other (the parasites), which is what would be expected if the two groups are locked in an evolutionary arms race. DNA-sequence data were collected for snails and parasites from the same localities. The findings indicated that coevolution was unlikely to have occurred. The implications of this are that host-switching or acquisition is more likely than previously thought. Consequently, there is a greater potential for spread of the parasite into new areas. The role of mountain barriers in confining schistosomiasis to certain regions was highlighted; this is important in view of the current plans to breach these barriers by road and rail construction that will link China and Southeast Asia.

## Introduction

### Background

In China schistosomiasis in humans is caused by infection with the parasitic blood-fluke *Schistosoma japonicum* (Trematoda: Digenea). Schistosomiasis causes major public health problems in China and the Philippines[1]. Despite over 45 years of integrated control efforts, approximately one million people, and more than 1.7 million bovines and other mammals, are currently infected in mainland China[2]. The disease develops after direct penetration of the skin by free-swimming parasite larvae (cercariae) released from the snail intermediate host. Exposure to cercariae of *S*. *japonicum* occurs through contact with wet vegetation or walking through rice fields or other flooded areas inhabited by infective snails. In the case of *S*. *japonicum*, only sub-species of the snail *Oncomelania hupensis* (Gastropoda: Pomatiopsidae) are able to act as intermediate host[3]. The snails become infected by similarly mobile and penetrative larvae (miracidia) passed in the feces of definitive hosts, which include a wide variety of mammals (up to 28 genera and 7 orders [1,4,5]).

Much of the transmission in mainland China occurs across the generally flat and marshy lake-land areas around the middle to lower Yangtze river, where *Oncomelania hupensis* is the snail intermediate host. In the highland areas of Southwest China (Sichuan and Yunnan) *O*. *hupensis robertsoni* is the intermediate host. Fewer people are infected in the highland areas; however, recrudescence is most marked in this region and in Sichuan between 1989 and 2004, the disease re-emerged in 8 counties (prevalence: 1.4% to 18.3% in humans, and up to 22.3% in cattle and 0.16% in snails)[6]. A third subspecies is found in China, *O*. *hupensis tangi*, which is endemic to hilly or coastal areas of Fujian, Guangdong and Guangxi provinces, although *S*. *japonicum* transmission appears to have been interrupted in these areas. In the Philippines, a further sub-species is responsible for transmission, namely *O*. *hupensis quadrasi*, and in Sulawesi *O*. *hupensis lindoensis* is responsible. Other subspecies exist in Taiwan and in Japan (where the only other species of *Oncomelania*, *O*. *minima*, is also found) but these are not, or are no longer, relevant to human health[7].

**Fig 1:**
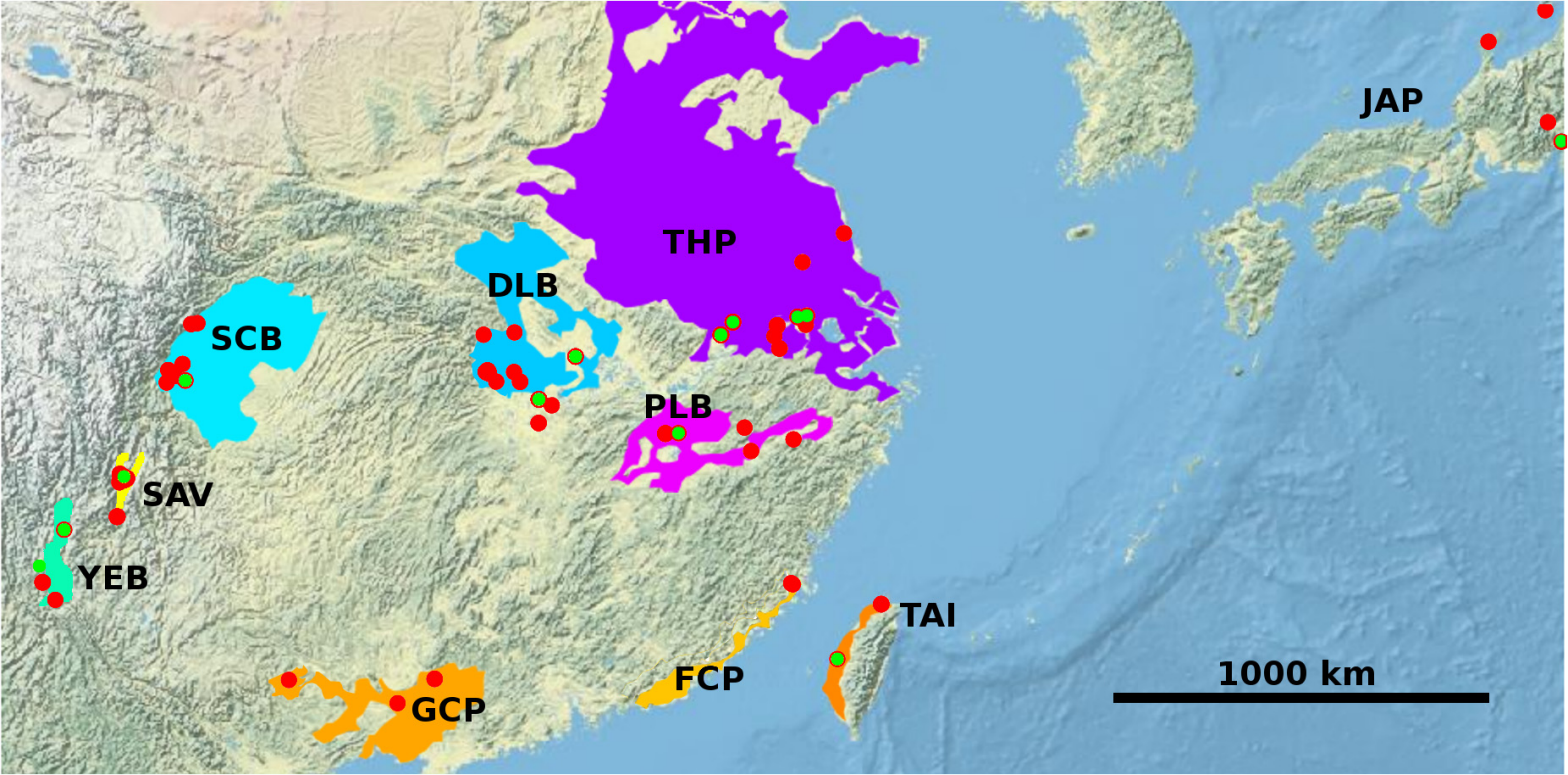
The locations of the sample sites in China and Japan. The coloured spots indicate areas from which snail and worm populations were sampled (red and green respectively). Most of the red spots lie on top of a green spot, indicating that the snails and the worms were sampled from the same locality. The coloured regions define biogeographical areas, within which there are no significant barriers to snail dispersal (e.g., no high mountains). Philippine samples (worms and snails) were included, but omitted from the map to increase resolution. Abbreviations are listed in Table 1. Plotted using the OpenStreetMap package in R.

Clearly, *S*. *japonicum* continues to pose a serious public health problem and inter-disciplinary research is required to understand the patterns of transmission and persistence of the disease. Phylogeographical studies can shed light on the problem, particularly in determining where the disease comes from, explaining current distributions of the intermediate host and predicting future epidemiology. Comparative phylogenetics can help detect patterns of host-parasite coevolution and indicate any potential for regional adaptation. Despite the potential of such approaches, relatively little work has been done in this area for *S*. *japonicum*. The present study was therefore performed in order to apply current phylodynamic techniques to the estimation of sources and tracts of dispersal for this parasite and to test for the signatures of host-parasite coevolution during the evolution of *S*. *japonicum* and its intermediate hosts (the latter necessarily having the defining influence on the distribution of the parasite). Strictly, the techniques used here test for phylogenetic congruence rather than directly for coevolution. The absence of phylogenetic congruence would make long-term coevolution unlikely; thus providing an indirect test for the latter. The study aimed to utilise the largest and most geographically extensive data set available (Fig 1).

**Table 1:** Malacological data used in the phylogeographical reconstructions–*cox*1 locus (CO1).

GAC	Taxon	Locality	Coordinates	Ref
Fujian coastal plain (China)—FCP				
DQ212796	*Oncomelania hupensis tangi*	Fuzhou, Fuqing, Donggang, Donghanzhen	25.430833 119.605278	[8]
JF284695	*Oncomelania hupensis tangi* *	Fuzhou, Fuqing, Donggang, Donghanzhen, Nansha	25.4049 119.6471	unpub
Guangxi coastal plain (China)—GCP				
DQ112264	*Oncomelania hupensis hupensis*	Xishanzhen, Guiping, Guigang (Yujiang river/Qian river)	23.391 110.078	*[9]*
DQ112265	*Oncomelania hupensis hupensis*	Xishanzhen, Guiping, Guigang (Yujiang river/Qian river)	23.391 110.078	*[9]*
DQ112266	*Oncomelania hupensis hupensis*	Xishanzhen, Guiping, Guigang (Yujiang river/Qian river)	23.391 110.078	*[9]*
GU367391	*Oncomelania hupensis*	Nanning, Taoxuzhen, Dingwu (Haitan Dao coastal plain W)	22.816667 109.116667	[10]
JF284696	*Oncomelania hupensis*	Nanning, Jing Xi Xian, Baise, Bameng Reservoir	23.3655 106.2997	unpub
Hunan / Hubei Dongting Lake Basin (China)—DLB				
AF253072	*Oncomelania hupensis hupensis*	Hubei, Wuhan, Caidian, Beiban (Houguan Hou)	30.569 114.017	*[8]*
AF253073	*Oncomelania hupensis hupensis*	Hubei, Wuhan, Caidian, Beiban (Houguan Hou)	30.569 114.017	*[8]*
AF254478	*Oncomelania hupensis hupensis*	Hubei, Wuhan, Caidian, Beiban (Houguan Hou)	30.569 114.017	[11]
AF254479	*Oncomelania hupensis hupensis*	Hubei, Wuhan, Caidian, Beiban (Houguan Hou)	30.569 114.017	[11]
AF254480-254489	*Oncomelania hupensis hupensis*	Hunan, Yue Yang, Junshan, Tangang Cun (Yangtze River)	29.617 113.067	[11]
AF306572-306581	*Oncomelania hupensis hupensis*	Hubei, Jingzhou, Songzi, Desheng Cun, Miao River (near Changhu)	30.20139 111.765028	[12]
AF306582	*Oncomelania hupensis hupensis*	Hubei, Jingzhou, Songzi, Qichansi, Miao River (nr Changhu)	30.25714 111.734641	[12]
AF306583	*Oncomelania hupensis hupensis*	Hubei, Jingzhou, Songzi, Qichansi, Miao River (nr Changhu)	30.25714 111.734641	[12]
AF306584	*Oncomelania hupensis hupensis*	Hubei, Jingzhou, Songzi, Qichansi, Miao River (nr Changhu)	30.25714 111.734641	[12]
AF306585	*Oncomelania hupensis hupensis*	Hubei, Jingzhou, Songzi, Qichansi, Miao River (nr Changhu)	30.25714 111.734641	[12]
AF306586	*Oncomelania hupensis hupensis*	Hubei, Jingzhou, Songzi, Qichansi, Miao River (nr Changhu)	30.25714 111.734641	[12]
AF306587	*Oncomelania hupensis hupensis*	Hubei, Jingzhou, Songzi, Qichansi, Miao River (nr Changhu)	30.25714 111.734641	[12]
AF306588-306597	*Oncomelania hupensis hupensis*	Hubei, Jingzhou, Songzi, Guanyue Cun, Miao River (near Changhu)	30.22888 111.692471	[12]
AF306598	*Oncomelania hupensis hupensis*	Hubei, Jingzhou, Songzi, Sunhe Supermarket Guanyue Cun	30.229074 111.691602	[12]
AF306599	*Oncomelania hupensis hupensis*	Hubei, Jingzhou, Songzi, Sunhe Supermarket Guanyue Cun	30.229074 111.691602	[12]
AF306600	*Oncomelania hupensis hupensis*	Hubei, Jingzhou, Songzi, Sunhe Supermarket Guanyue Cun	30.229074 111.691602	[12]
AF306601	*Oncomelania hupensis hupensis*	Hubei, Jingzhou, Songzi, Sunhe Supermarket Guanyue Cun	30.229074 111.691602	[12]
AF306602	*Oncomelania hupensis hupensis*	Hubei, Jingzhou, Songzi, Sunhe Supermarket Guanyue Cun	30.229074 111.691602	[12]
AF306603-306612	*Oncomelania hupensis hupensis*	Hubei, Jingzhou, Songzi, Longtanhe Cun, Miao River (near Changhu)	30.230595 111.677649	[12]
AF306613-306621	*Oncomelania hupensis hupensis*	Hubei, Jingzhou, Songzi, Wangjia Dayuan, Miao River (near Changhu)	30.22455 111.754768	[12]
AF306622-306630	*Oncomelania hupensis hupensis*	Hubei, Jingzhou, Songzi, Huayuanwu, Miao River (near Changhu)	30.24713 111.742787	[12]
DQ112267	*Oncomelania hupensis hupensis*	Hubei, Jingzhou, Jiangling, Xiaojiaju (Yangtze River)	30.009 112.579	[8]
DQ112268	*Oncomelania hupensis hupensis*	Hubei, Jingzhou, Jiangling, Xiaojiaju (Yangtze River)	30.009 112.579	[8]
EU001660	*Oncomelania hupensis hupensis*	Hubei, Yudian River, Wuhan, Suizhou, Huangjiawan	31.839056 113.681889	[13]
EU333403	*Oncomelania hupensis hupensis*	Wuhan, Jingmen,Liujialing (near Nanhu lake & Hanshui river)	31.1 112.433333	[10]
FJ997214	*Oncomelania hupensis hupensis*	Hunan, Yueyang, Zhongzhouxiang (Xiajiang river)	29.0861 113.0595	[14]
GU367390	*Oncomelania hupensis hupensis*	Hunan, Yue Yang, Linxiang, Baiyun	29.483333 113.4	[10]
GU367392	*Oncomelania hupensis hupensis*	Hubei, Yichang, Yuan An Xian, Xiangjiapo (Juhe river)	31.05 111.633333	[10]
JF284689	*Oncomelania hupensis hupensis*	Hubei, Jingzhou, Shizikouzhen, Qunxing	30.0127 111.9602	unpub
JF284690	*Oncomelania hupensis hupensis*	Hubei, Jingzhou, Jiangling, Gejiatai, Zhujianao	30.2261 112.4206	unpub
JF284692	*Oncomelania hupensis hupensis*	Hunan, Yue Yang, Zhongzhouxiang (Xiajiang river)	29.0861 113.0595	unpub
Mid-Yangtze Poyang Lake Basin (China)—PLB				
**KR002674**	***Oncomelania hupensis hupensis***	**Jiangxi, Jiangshan, Xianyanzhen, Houcang**	**28.590853,118.370476**	
DQ112255-112263	*Oncomelania hupensis hupensis*	Jiangxi, Jiujiang, Pengze (Yangtze river/Huanghu near Poyang Lake)	28.983 116.15	*[9]*
GU367393	*Oncomelania hupensis hupensis*	Zhejiang, Quzhou, Kaihua, Chihuaizhen, Youchuan Cun (Huangshan)	29.116667 118.2	[10]
JF284693	*Oncomelania hupensis hupensis*	Jiangxi, Jiujiang, Jingdezhen, Zhongshanpo (Daming Lake)	28.9931 116.4887	unpub
JF284694	*Oncomelania hupensis hupensis*	Zhejiang, Quzhou, Jinhua, Liugoukou, Shafan Reservoir	28.8523 119.4688	unpub
Mid-Yangtze to Lower Yangtze Taihu Plain (China)—THP				
AF254490-254499	*Oncomelania hupensis hupensis*	Anhui, Ning Guo County	30.3730 118.9725	[11]
AF254500-254509	*Oncomelania hupensis hupensis*	Anhui, Chizhou, Guichi, Chikou	30.6734 117.4549	[11]
AF254510	*Oncomelania hupensis hupensis*	Zhejiang, Guangde, Guzhushan, Houchong (near Taihu)	31.0931 119.685	[11]
AF254511	*Oncomelania hupensis hupensis*	Zhejiang, Guangde, Guzhushan, Houchong (near Taihu)	31.0931 119.685	[11]
AF254512	*Oncomelania hupensis hupensis*	Zhejiang, Guangde, Guzhushan, Houchong (near Taihu)	31.0931 119.685	[11]
AF254513	*Oncomelania hupensis hupensis*	Zhejiang, Guangde, Guzhushan, Houchong (near Taihu)	31.0931 119.685	[11]
AF254514	*Oncomelania hupensis hupensis*	Zhejiang, Guangde, Guzhushan, Houchong (near Taihu)	31.0931 119.685	[11]
AF254515	*Oncomelania hupensis hupensis*	Zhejiang, Guangde, Guzhushan, Houchong (near Taihu)	31.0931 119.685	[11]
AF254516-254525	*Oncomelania hupensis hupensis*	Anhui, Guangde County, Dongjia Datang, Xinhangzhen	31.0490 119.550	[11]
AF254526	*Oncomelania hupensis hupensis*	Jiangsu, XiaShesizu, WangTaosizu, Dongtai, Yancheng, coastal	32.8911 120.6460	[11]
AF254527	*Oncomelania hupensis hupensis*	Jiangsu, XiaShesizu, WangTaosizu, Dongtai, Yancheng, coastal	32.8911 120.6460	[11]
AF254528	*Oncomelania hupensis hupensis*	Jiangsu, XiaShesizu, WangTaosizu, Dongtai, Yancheng, coastal	32.8911 120.6460	[11]
AF254529	*Oncomelania hupensis hupensis*	Jiangsu, XiaShesizu, WangTaosizu, Dongtai, Yancheng, coastal	32.8911 120.6460	[11]
AF254530	*Oncomelania hupensis hupensis*	Jiangsu, XiaShesizu, WangTaosizu, Dongtai, Yancheng, coastal	32.8911 120.6460	[11]
AF254531	*Oncomelania hupensis hupensis*	Jiangsu, XiaShesizu, WangTaosizu, Dongtai, Yancheng, coastal	32.8911 120.6460	[11]
AF254532	*Oncomelania hupensis hupensis*	Zhejiang, Huzhou, Guangde, Xianshanhu, Si'anzhen	30.8955 119.6562	[11]
AF254533	*Oncomelania hupensis hupensis*	Zhejiang, Huzhou, Guangde, Xianshanhu, Si'anzhen	30.8955 119.6562	[11]
AF254534	*Oncomelania hupensis hupensis*	Zhejiang, Huzhou, Guangde, Xianshanhu, Si'anzhen	30.8955 119.6562	[11]
AF254535	*Oncomelania hupensis hupensis*	Zhejiang, Huzhou, Guangde, Xianshanhu, Si'anzhen	30.8955 119.6562	[11]
AF254536	*Oncomelania hupensis hupensis*	Anhui, Tongling, Hujiamen, Guanghui Cun, LaoDao Island	30.9542 117.7664	[11]
AF254537	*Oncomelania hupensis hupensis*	Anhui, Tongling, Hujiamen, Guanghui Cun, LaoDao Island	30.9542 117.7664	[11]
AF254538	*Oncomelania hupensis hupensis*	Anhui, Tongling, Hujiamen, Guanghui Cun, LaoDao Island	30.9542 117.7664	[11]
AF254539	*Oncomelania hupensis hupensis*	Anhui, Tongling, Hujiamen, Guanghui Cun, LaoDao Island	30.9542 117.7664	[11]
AF254540	*Oncomelania hupensis hupensis*	Anhui, Tongling, Hujiamen, Guanghui Cun, LaoDao Island	30.9542 117.7664	[11]
AF254541	*Oncomelania hupensis hupensis*	Anhui, Tongling, Hujiamen, Guanghui Cun, LaoDao Island	30.9542 117.7664	[11]
AF254542-254555	*Oncomelania hupensis hupensis*	Anhui, Xuancheng, Sunbuzhen, Mawang Cun	30.8780 118.9143	[11]
JF284686	*Oncomelania hupensis hupensis*	Anhui, Huzhou, Guangde, Yinjiwan, Jingliu Cun (Taihu lake)	31.0675 119.4432	unpub
JF284687	*Oncomelania hupensis hupensis*	Anhui, Guangde, Shanbeixiang, Jishan Cun, Nianzhiwu	31.0675 119.4432	unpub
JF284688	*Oncomelania hupensis hupensis*	Jiangsu, Yangzhou, Wantou (Yangtze river)	32.2651 119.5707	unpub
Sichuan: Anning River Valley (China)—SAV				
AF213339	*Oncomelania hupensis robertsoni*	Liangshan, Shizuizi	27.84284 102.39277	[15]
DQ112250	*Oncomelania hupensis robertsoni*	Liangshan, Miyi, Hongshiyan, Guanyinxiang, Shujingwan	26.963611 102.132778	*[9]*
DQ112251	*Oncomelania hupensis robertsoni*	Liangshan, Miyi, Hongshiyan, Guanyinxiang, Shujingwan	26.963611 102.132778	*[9]*
DQ212814-212820	*Oncomelania hupensis robertsoni*	Liangshan, Xixiangxiang, Gucheng Cun	27.93525 102.20540	[8]
DQ212821	*Oncomelania hupensis robertsoni*	Liangshan (near Qionghai sea) Yanjia Buzi (Heyan Road)	27.87505 102.30867	[8]
DQ212822	*Oncomelania hupensis robertsoni*	Liangshan (near Qionghai sea) Yanjia Buzi (Heyan Road)	27.87505 102.30867	[8]
DQ212823	*Oncomelania hupensis robertsoni*	Liangshan (near Qionghai sea) Yanjia Buzi (Heyan Road)	27.87505 102.30867	[8]
DQ212824	*Oncomelania hupensis robertsoni*	Liangshan (near Qionghai sea) Yanjia Buzi (Heyan Road)	27.87505 102.30867	[8]
DQ212825	*Oncomelania hupensis robertsoni*	Liangshan off left side of G5 Jingku Gao Su, Jingjiuxiang	27.8000 102.204	[8]
DQ212826	*Oncomelania hupensis robertsoni*	Liangshan off left side of G5 Jingku Gao Su, Jingjiuxiang	27.8000 102.204	[8]
DQ212827	*Oncomelania hupensis robertsoni*	Liangshan off left side of G5 Jingku Gao Su, Jingjiuxiang	27.8000 102.204	[8]
DQ212828	*Oncomelania hupensis robertsoni*	Liangshan off left side of G5 Jingku Gao Su, Jingjiuxiang	27.8000 102.204	[8]
DQ212829	*Oncomelania hupensis robertsoni*	Liangshan Qionghai Lake, Hainanxiang	27.7995 102.3087	[8]
DQ212830	*Oncomelania hupensis robertsoni*	Liangshan Qionghai Lake, Hainanxiang1	27.7995 102.3087	[8]
DQ212831	*Oncomelania hupensis robertsoni*	Liangshan Qionghai Lake, Hainanxiang1	27.7995 102.3087	[8]
DQ212832	*Oncomelania hupensis robertsoni*	Liangshan Qionghai Lake, Hainanxiang1	27.7995 102.3087	[8]
DQ212833	*Oncomelania hupensis robertsoni*	Liangshan Qionghai Lake, Hainanxiang2	27.7973 102.3157	[8]
DQ212834	*Oncomelania hupensis robertsoni*	Liangshan Qionghai Lake, Hainanxiang2	27.7973 102.3157	[8]
DQ212835	*Oncomelania hupensis robertsoni*	Liangshan Qionghai Lake, Hainanxiang2	27.7973 102.3157	[8]
DQ212836	*Oncomelania hupensis robertsoni*	Liangshan Qionghai Lake, Hainanxiang2	27.7973 102.3157	[8]
DQ212837	*Oncomelania hupensis robertsoni*	Liangshan G 5 Jing Kun Gao Su Chu Kou, Dafenba	27.7468 102.1903	[8]
DQ212838	*Oncomelania hupensis robertsoni*	Liangshan G 5 Jing Kun Gao Su Chu Kou, Dafenba	27.7468 102.1903	[8]
DQ212839	*Oncomelania hupensis robertsoni*	Liangshan G 5 Jing Kun Gao Su Chu Kou, Dafenba	27.7468 102.1903	[8]
DQ212840	*Oncomelania hupensis robertsoni*	Liangshan G 5 Jing Kun Gao Su Chu Kou, Dafenba	27.7468 102.1903	[8]
DQ212841-212851	*Oncomelania hupensis robertsoni*	Liangshan, Miyi, G5 Jingkun expressway, Shujingwan	26.9637 102.1328	[8]
EU079378	*Oncomelania hupensis robertsoni*	Liangshan, Mazengyiwuxiang, Weija Diaolou	27.817833 102.369472	[13]
JF284691	*Oncomelania hupensis robertsoni*	Liangshan, Erwu, Yiwanshui	27.8773 102.3703	unpub
Sichuan Plain (China)—SCB				
**KR002675**	***Oncomelania hupensis robertsoni***	**Chengdu Xinjin**	**30.405452,103.829727**	
AF531547	*Oncomelania hupensis robertsoni*	Mianzhu, Tiezhuangyan (Mawei River)	31.302792 104.215357	[16]
DQ212797	*Oncomelania hupensis robertsoni*	Meishan, Danling, Xindianzi, Qinglongzui	29.99163 103.41580	[8]
DQ212798	*Oncomelania hupensis robertsoni*	Meishan, Danling, Xindianzi, Qinglongzui	29.99163 103.41580	[8]
DQ212799-212808	*Oncomelania hupensis robertsoni*	Meishan Dongpo Pan'aoxiang Wangshigou	30.13993 103.61167	[8]
DQ212809	*Oncomelania hupensis robertsoni*	Meishan, Dongpo, Baiyang Cun	30.0373 103.9002	[8]
DQ212810	*Oncomelania hupensis robertsoni*	Meishan, Dongpo, Baiyang Cun	30.0373 103.9002	[8]
DQ212811	*Oncomelania hupensis robertsoni*	Meishan, Dongpo, Baiyang Cun	30.0373 103.9002	[8]
DQ212812	*Oncomelania hupensis robertsoni*	Meishan, Dongpo, Baiyang Cun	30.0373 103.9002	[8]
DQ212813	*Oncomelania hupensis robertsoni*	Meishan, Dongpo, Baiyang Cun	30.0373 103.9002	[8]
JF284697	*Oncomelania hupensis robertsoni*	Ya'an, Qionglai, Daxingzhen, Shuikou Cun, Xucitang	30.2701 103.4562	unpub
Taiwan (China)—TAI				
DQ112271-112280	*Oncomelania hupensis chiui*	Taipei, Shimen, Alilao Cun (Coastal)	25.278607 121.61164	unpub
DQ112281	*Oncomelania hupensis formosana*	Taiwan (W) Changhua, Puyan Township	23.996416 120.475913	[8]
DQ112282	*Oncomelania hupensis formosana*	Taiwan (W) Changhua, Puyan Township	23.996416 120.475913	[8]
DQ112283	*Oncomelania hupensis formosana*	Taiwan (W) Changhua, Puyan Township	23.996416 120.475913	[8]
EF394891	*Oncomelania hupensis chiui*	Taipei, Shimen, Alilao Cun (Coastal)	25.279121 121.611056	[17]
Yunnan: Erhai Basin—YEB				
AF253074	*Oncomelania hupensis robertsoni*	Dali, Tongsipan	25.4517 100.2011	unpub
AF253075	*Oncomelania hupensis robertsoni*	Dali, Tongsipan	25.4517 100.2011	unpub
DQ112252	*Oncomelania hupensis robertsoni*	Dali, Yongjianzhen, Tongsipan	25.4510 100.2007	[8]
DQ112253	*Oncomelania hupensis robertsoni*	Dali, Yongjianzhen, Tongsipan	25.4510 100.2007	[8]
DQ112254	*Oncomelania hupensis robertsoni*	Dali, Yongjianzhen, Tongsipan	25.4510 100.2007	[8]
DQ212852	*Oncomelania hupensis robertsoni*	Dali, Yongjianzhen, Tongsipan	25.4510 100.2007	[8]
DQ212853	*Oncomelania hupensis robertsoni*	Dali, Yongjianzhen, Tongsipan	25.4510 100.2007	[8]
DQ212854	*Oncomelania hupensis robertsoni*	Dali, Yongjianzhen, Tongsipan	25.4510 100.2007	[8]
DQ212855	*Oncomelania hupensis robertsoni*	Dali, Yongjianzhen, Tongsipan	25.4510 100.2007	[8]
DQ212856	*Oncomelania hupensis robertsoni*	Dali, Yongjianzhen, Tongsipan	25.4510 100.2007	[8]
DQ212857	*Oncomelania hupensis robertsoni*	Dali, Yongjianzhen, Tongsipan	25.4510 100.2007	[8]
EU688958	*Oncomelania hupensis robertsoni*	Dali, Nanjianzhen	25.043391 100.53031	unpub
EU688959	*Oncomelania hupensis robertsoni*	Lijiang, Yongsheng	26.664488 100.758963	unpub
EU780225	*Oncomelania hupensis robertsoni*	Dali, Chao Yang Cun	25.82887 100.11777	unpub
GU367389	*Oncomelania hupensis robertsoni*	Lijiang, Yongsheng	26.683333 100.75	unpub
Japan—JAP				
**KR002673**	***Oncomelania hupensis nosophora***	**Honshu, Chiba Kisarazu**	**35.350371 139.988407**	
AB611787	*Oncomelania hupensis nosophora*	Honshu, Yamanashi, Nirasaki	35.715 138.434	[18]
AB611791	*Oncomelania minima*	Honshu, Ishikawa, Wajima	37.376 136.894	[18]
AB611795	*Oncomelania minima*	Sado Island, Niigata	38.014 138.368	[18]
DQ112284	*Oncomelania hupensis nosophora*	Honshu Kanagawa Kiyokawa	35.3976 139.9533	[8]
DQ112285	*Oncomelania hupensis nosophora*	Honshu Kanagawa Kiyokawa	35.3976 139.9533	[8]
DQ112286	*Oncomelania hupensis nosophora*	Honshu Kanagawa Kiyokawa	35.3976 139.9533	[8]
Philippines—PHL				
JF284698	*Oncomelania hupensis quadrasi* *	Mindoro, Mansalay	12.547 121.442	[19]
DQ112289	*Oncomelania hupensis quadrasi*	Leyte, Palo	11.16 124.98	unpub
DQ112287	*Oncomelania hupensis quadrasi*	Leyte, Palo	11.16 124.98	unpub
DQ112288	*Oncomelania hupensis quadrasi*	Leyte, Palo	11.16 124.98	unpub

Data are: GenBank accession numbers (GACs), collection localities (with coordinates and region code as used in Fig 1, e.g., FCP) and (Ref) source references (citations in italics refer to locality information only and not to the publication of the GAC; unpub, indicates unpublished data with locations drawn from isolate codes, specimen voucher numbers or details in the GenBank). Taxonomic nomenclature follows[9]. Data collected as part of the present study are marked in bold.

*Incorrectly listed as Chinese *Oncomelania hupensis hupensis* on GenBank

A geographical strain complex has been revealed within *Schistosoma japonicum* using partial DNA sequences of mitochondrial genes [22,23]. In 2005 microsatellites were used to assess variation among *S*. *japonicum* populations from 8 geographical locations in 7 endemic provinces across mainland China. The populations were found to fall into two main clades, a Sichuan/Yunnan clade and a lake/marshland clade of the middle and lower Yangtze river drainage[24]. The finding was later corroborated[25] using partial mitochondrial gene sequences (*cytb*-*nd4*L-*nd4*, *cox3*, *nad1 nad4*, *nad5* and 16S-12S), with the additional observation that the lake/marshland clade included a highly diverse lower Yangtze sub-clade[25,26]. Molecular phylogenetic studies of *Oncomelania hupensis* are relatively few in number, but a major study in 2009[19] suggested that *O*. *hupensis* populations across China fall into three main clades. These were a middle/lower Yangtze lake and a marshland clade (*O*. *hupensis hupensis*), a Sichuan/Yunnan mountain clade (*O*. *h*. *robertsoni*), a clade corresponding to populations of the hilly littoral areas of Fujian (*O*. *h*. *tangi*) and a clade of the karst region of Guangxi (*O*. *h*. *hupensis* or, according to the 2009 authors, *O*. *h*. *guangxiensis*). A more recent study used complete mitochondrial genome sequences to obtain mean genetic distance estimate of 12% for the protein coding genes between *O*. *h*. *hupensis* and *O*. *h*. *robertsoni*[13]. A study with similarly wide geographical coverage to that used here has been performed[27]; this employed DNA sequence variation in *O*. *hupensis* to indicate that isolation by distance was more significant in shaping genetic divergence than isolation by environment; however, this study which covered 29 localities, was population genetic (not comparative phylogenetic) and did not include the parasite.

### Aims of the study

Although there have been past population phylogenetic studies of both *Oncomelania* and *S*. *japonicum* none has used as geographically diverse, taxon and character rich, data set as the present study. Also, this is the first quantitative comparison of the phylogenetic history of the snails and the parasites. Relatively few phylogenetic studies have considered the origins of these taxa, although Davis[28] proposed a Gondwanan origin for the Pomatiopsidae (including *Oncomelania* and proto-*S*. *japonicum*), with rafting to mainland Asia (via the Indian Craton after the break up of Gondwana) and colonisation of Southeast Asia and China (Tibet/Yunnan) via the northern-India-Myanmar, Brahmaputra-Irrawaddy river corridor in an West to East direction, around 18 Ma. In contrast, an East to West hypothesis has been proposed for the Chinese Pomatiopsidae taxa[29], with precursors of *Oncomelania* colonising originally from Australasia and via the Philippines, along island chains created by low sea levels and by tectonic movements (rafting). After reaching Japan, Proto-*Oncomelania* gives rise to *Oncomelania hupensis* in mainland China; the latter then recolonises Japan, the Philippines and Sulawesi (replacing antecedent forms). In a recent paper[30], the East to West hypothesis was tested and the radiation across China dated at 15–5 Ma (by molecular clock); however, the radiation of *S*. *japonicum* did not appear to be isochronous with that of the present day intermediate hosts[31,32]. Nevertheless, the history of host utilization in *Schistosoma* has been regarded as an evolutionary battle with snail defences[11,33–37], with the schistosome under significant pressure to evolve counter measures to snail immune responses, or to track snail divergence in an evolutionary arms race (the ‘Red Queen hypothesis’[38]).

Schistosomiasis researchers postulating coevolution have evidenced this by citing restriction of the parasites to certain snail lineages[11], high levels of genetic variation in naturally exposed snail populations[34], and evidence for selection in schistosomes exposed to snails previously selected for resistance[36]. The latter study[36] did use genetic (microsatellite) variation to demonstrate higher rates of parasite divergence in resistant laboratory lines of snails; however, this was over a very short time-scale and the resistant populations had not evolved under natural conditions. The study also used the *Schistosoma mansoni*:*Biomphalaria glabrata* system (which involves far higher infection rates than seen with *S*. *japonicum–*see Discussion). Consequently, further studies are needed into the role of coevolution in the evolutionary history of *Schistosoma*.

The present investigation was performed in view of the lack of studies on *S*. *japonicum* comparing the parasite and snail intermediate host evolutionary histories, the alternative hypotheses regarding the origins of these taxa and the colonisation of mainland China, and the recent availability of additional DNA sequence data (improving geographical coverage). It is important to address these questions because they can help explain the current distribution of the parasite within the range of the snails, which is relevant to snail control strategies and the potential for range expansion, and to help assess the likely impacts of environmental manipulation such as the South-to-North-Water-Transfer project in China and the construction of road links. The Greater Mekong Subregion (GMS) Chengdu-Kunming corridor and GMS North-South Corridor from Yunnan, through Laos and to Thailand (NSEC1), both involve tunnels and rapid links through the mountains of Sichuan and Yunnan to Laos[39]. Consequently, the work will not only improve our understanding of host-parasite coevolution, but also shed light on the impacts of development in the region.

## Methods

### Sampling

Three new samples of *Oncomelania* were taken and sequenced for this study; these were *O*. *hupensis* from Jiangshan in Jiangxi Province, China (*cox*1), and from Xinjin in Chengdu, Sichuan Province, China (*cox*1 and *rrn*L), and *O*. *hupensis nosophora* from Kanagawa Kiyokawa in Honshu, Japan (*cox*1 and *rrn*L)–these loci all belonging to the mitochondrial genome, with partial DNA sequences of each locus obtained using the popular Folmer[40] and Palumbi[41] primers, to allow comparisons with data from earlier studies. The three samples were identified on the basis of conchology, morphology and ecological habit (following[9]). The identifications were performed by YS and NH, two greatly experienced medical malacologists. The DNA sequencing followed procedures detailed elsewhere[30]. The remainder of the data were obtained from GenBank to give a total of 265 *cox*1 sequences and 70 *rrn*L sequences, apparently orthologous with the new sequence data above. Sequence data for *Tricula hortensis* from Sichuan China, also a pomatiopsid snail[42], were included for use as an outgroup. In addition to the data for *Oncomelania*, 14 complete *cox*1 and *cox*3 DNA sequences were obtained from the GenBank for samples from 14 *Schistosoma japonicum* transmission localities across China. A corresponding *Schistosoma mekongi* sequence was obtained as an outgroup taxon for the *S*. *japonicum* analyses. Full details of the data used and sampling range are given in Fig 1 and Tables 1, 2 and 3. The data set included all available relevant sequence data in the GenBank at the time of searching; however, some data (10 sequences) were excluded because of uncertain origin (e.g., DQ112269, DQ112270) and taxonomy (e.g., EU780224). The *cox*1 and *cox*3 loci were chosen following earlier work, which indicated that, for *Schistosoma*, *cox*3 was the locus of choice in terms of consistent phylogenetic signal and sufficient number of phylogenetically informative characters per site; this was followed by *nad*4, however, the present data showed a haplotype diversity <1 at this locus and *cox*1 was used because it gave high support values and “correct” topology in the same study[43]. Many of the localities listed in Tables 1 and 2 were not published either in GenBank or in the papers where the data were first presented. In these cases the localities were found by accessing field work reports cited in the paper presenting the sequence data (if present), matching published sample codes with associated accession numbers, reference to museum specimen accession numbers, contacting original researchers (or the local officials who accompanied them or those who arranged their collections) and referring to the personal observations of the present authors. In some cases, incomplete (e.g., to County level only) location data was given, or ambiguously transliterated place names, in these cases location records were completed and transliterations resolved. The locations in Tables 1 and 2 have been changed (where necessary) to use the closest place name (village etc) appearing on Google Maps; this is for the convenience of future authors.

**Table 2:** Malacological data used in the phylogeographical reconstructions–*rrn*L locus (16S).

GAC		Taxon	Locality	Coordinates	Ref
Fujian coastal plain (China)—FCP						
DQ212860		*Oncomelania hupensis tangi*		Fuzhou, Fuqing, Donggang, Donghanzhen	25.430833, 119.605278	[8]
JF284695		*Oncomelania hupensis tangi* *		Fuzhou, Fuqing, Donggang, Donghanzhen, Nansha	25.4049 119.6471	unpub
Guangxi coastal plain (China)—GCP						
JF284696		*Oncomelania hupensis*		Nanning, Jing Xi Xian, Baise, Bameng Reservoir	23.3655 106.2997	unpub
Hunan / Hubei Dongting Lake Basin (China)—DLB						
EU001660		*Oncomelania hupensis hupensis*		Hubei, Yudian River, Wuhan, Suizhou, Huangjiawan	30.189056 112.181889	[13]
FJ997214		*Oncomelania hupensis hupensis*		Hunan, Yueyang, Zhongzhouxiang (Xiajiang river)	29.0861 113.0595	[14]
JF284689		*Oncomelania hupensis hupensis*		Hubei, Jingzhou, Shizikouzhen, Qunxing	30.0127 111.9602	unpub
JF284690		*Oncomelania hupensis hupensis*		Hubei, Jingzhou, Jiangling, Gejiatai, Zhujianao	30.2261 112.4206	unpub
JF284692		*Oncomelania hupensis hupensis*		Hunan, Yue Yang, Zhongzhouxiang (Xiajiang river)	29.0861 113.0595	unpub
Mid-Yangtze Poyang Lake Basin (China)—PLB						
JF284687		*Oncomelania hupensis hupensis*		Anhui, Guangde, Shanbeixiang, Jishan Cun, Nianzhiwu	31.0675 119.4432	unpub
JF284693		*Oncomelania hupensis hupensis*		Jiangxi, Jiujiang, Jingdezhen, Zhongshanpo (Daming Lake)	28.9931 116.4887	unpub
JF284694		*Oncomelania hupensis hupensis*		Zhejiang, Quzhou, Jinhua, Liugoukou, Shafan Reservoir	28.8523 119.4688	unpub
Mid-Yangtze to Lower Yangtze Taihu Plain (China)—THP						
DQ21259		*Oncomelania hupensis hupensis*		Wuhu, Xuancheng, Ningguo, Jiangjiashan	30.641921 118.841993	[8]
JF284686		*Oncomelania hupensis hupensis*		Anhui, Huzhou, Guangde, Yinjiwan, Jingliu Cun (Taihu lake)	31.0675 119.4432	unpub
JF284688		*Oncomelania hupensis hupensis*		Jiangsu, Yangzhou, Wantou (Yangtze river)	32.2651 119.5707	unpub
Sichuan: Anning River Valley (China)—SAV						
AF212893		*Oncomelania hupensis robertsoni*		Liangshan, Shizuizi	27.84284 102.39277	[15]
DQ212872		*Oncomelania hupensis robertsoni*		Liangshan, Xixiangxiang, Gucheng Cun	27.93525 102.20540	[8]
DQ212873		*Oncomelania hupensis robertsoni*		Liangshan Qionghai Lake, Hainanxiang	27.7973 102.3157	[8]
DQ212874		*Oncomelania hupensis robertsoni*		Liangshan, Xixiangxiang, Gucheng Cun	27.93525 102.20540	[8]
DQ212875		*Oncomelania hupensis robertsoni*		Liangshan, Xixiangxiang, Gucheng Cun	27.93525 102.20540	[8]
DQ212876		*Oncomelania hupensis robertsoni*		Liangshan (near Qionghai sea) Yanjia Buzi (Heyan Road)	27.87505 102.30867	[8]
DQ212877		*Oncomelania hupensis robertsoni*		Liangshan (near Qionghai sea) Yanjia Buzi (Heyan Road)	27.87505 102.30867	[8]
DQ212878		*Oncomelania hupensis robertsoni*		Liangshan (near Qionghai sea) Yanjia Buzi (Heyan Road)	27.87505 102.30867	[8]
DQ212879		*Oncomelania hupensis robertsoni*		Liangshan (near Qionghai sea) Yanjia Buzi (Heyan Road)	27.87505 102.30867	[8]
DQ212880		*Oncomelania hupensis robertsoni*		Liangshan off left side of G5 Jingku Gao Su, Jingjiuxiang	27.8000 102.204	[8]
DQ212881		*Oncomelania hupensis robertsoni*		Liangshan off left side of G5 Jingku Gao Su, Jingjiuxiang	27.8000 102.204	[8]
DQ212882		*Oncomelania hupensis robertsoni*		Liangshan off left side of G5 Jingku Gao Su, Jingjiuxiang	27.8000 102.204	[8]
DQ212883		*Oncomelania hupensis robertsoni*		Liangshan off left side of G5 Jingku Gao Su, Jingjiuxiang	27.8000 102.204	[8]
DQ212884		*Oncomelania hupensis robertsoni*		Liangshan Qionghai Lake, Hainanxiang1	27.7995 102.3087	[8]
DQ212885		*Oncomelania hupensis robertsoni*		Liangshan Qionghai Lake, Hainanxiang1	27.7995 102.3087	[8]
DQ212886		*Oncomelania hupensis robertsoni*		Liangshan Qionghai Lake, Hainanxiang1	27.7995 102.3087	[8]
DQ212887		*Oncomelania hupensis robertsoni*		Liangshan Qionghai Lake, Hainanxiang2	27.7973 102.3157	[8]
DQ212888		*Oncomelania hupensis robertsoni*		Liangshan Qionghai Lake, Hainanxiang2	27.7973 102.3157	[8]
DQ212889		*Oncomelania hupensis robertsoni*		Liangshan G 5 Jing Kun Gao Su Chu Kou, Dafenba	27.7468 102.1903	[8]
DQ212890		*Oncomelania hupensis robertsoni*		Liangshan G 5 Jing Kun Gao Su Chu Kou, Dafenba	27.7468 102.1903	[8]
DQ212891-212898		*Oncomelania hupensis robertsoni*		Liangshan, Miyi, G5 Jingkun expressway, Shujingwan	26.9637 102.1328	[8]
EU079378		*Oncomelania hupensis robertsoni*		Liangshan, Mazengyiwuxiang, Weija Diaolou	27.817833 102.369472	[13]
JF284691		*Oncomelania hupensis robertsoni*		Liangshan, Erwu, Yiwanshui	27.8773 102.3703	unpub
Sichuan Plain (China)—SCB						
**KR002677**		***Oncomelania hupensis robertsoni***		**Chengdu Xinjin**	**30.405452 103.829727**	
AF531545		*Oncomelania hupensis robertsoni*		Mianzhu, Tiezhuangyan (Mawei River)	31.302792 104.215357	[16]
DQ212863-212871		*Oncomelania hupensis robertsoni*		Meishan Dongpo Pan'aoxiang Wangshigou	30.1399 103.6117	[8]
JF284697		*Oncomelania hupensis robertsoni*		Ya'an, Qionglai, Daxingzhen, Shuikou Cun, Xucitang	30.2701 103.4562	unpub
Taiwan (China)—TAI						
DQ212861		*Oncomelania hupensis formosana*		Taiwan (W) Changhua, Puyan Township	23.996416 120.475913	[8]
Yunnan: Erhai Basin—YEB						
DQ212899		*Oncomelania hupensis robertsoni*		Dali, Yongjianzhen, Tongsipan	25.4510 100.2007	[8]
DQ212900		*Oncomelania hupensis robertsoni*		Dali, Yongjianzhen, Tongsipan	25.4510 100.2007	[8]
DQ212901		*Oncomelania hupensis robertsoni*		Dali, Yongjianzhen, Tongsipan	25.4510 100.2007	[8]
DQ212902		*Oncomelania hupensis robertsoni*		Dali, Yongjianzhen, Tongsipan	25.4510 100.2007	[8]
Japan—JAP						
**KR002676**		***Oncomelania hupensis nosophora***		**Honshu, Chiba Kisarazu**	**35.350371 139.988407**	
AB611786		*Oncomelania hupensis nosophora*		Honshu, Yamanashi, Nirasaki	35.715 138.434	[18]
AB611790		*Oncomelania minima*		Honshu, Ishikawa, Wajima	37.376 136.894	[18]
AB611794		*Oncomelania minima*		Sado Island, Niigata	38.014 138.368	[18]
DQ212858		*Oncomelania minima*		Honshu, Tokyo Chiyoda	35.6862 139.7534	[8]
Philippines—PHL						
JF284698		*Oncomelania hupensis quadrasi* *		Mindoro, Mansalay	12.547 121.442	unpub
DQ212862		*Oncomelania hupensis quadrasi*		Luzon, Tarlac, Victoria, Calibungan	15.5930 120.7388	[8]

Details as Table 1.

*Incorrectly listed as Chinese *Oncomelania hupensis hupensis* on GenBank

**Table 3:** Parasite data used in the phylogeographical reconstructions.

GAC		Sampling method	Locality	Coordinates		Ref
Hunan / Hubei Dongting Lake Basin(China)—DLB						
HM120842	FCC		Hubei, Wuhan, Caidian, Beiban (Houguan Hou)		30.569 114.017	[20]
HM120845	FCC		Hunan, Yue Yang, Junshan, Tangang Cun (Yangtze River)		29.617 113.067	[20]
Mid-Yangtze Poyang Lake Basin (China)—PLB						
HM120844	FCC		Jiangxi, Jiujiang, Jingdezhen, Zhongshanpo (Daming Lake)		28.9931 116.4887	[20]
Mid-Yangtze to Lower Yangtze Taihu Plain (China)—THP						
HM120843	FCC		Zhejiang, Guangde, Guzhushan, Houchong (near Taihu)		31.0931 119.6850	[20]
NC002544	ADL		Anhui, Tongling, Hujiamen, Guanghui Cun, LaoDao Island		30.9542 117.7664	[21]
KF279405-6	ADL		Anhui, Chizhou, Guichi, Chikou		30.6734 117.4549	unpub
HM120841	FCC		Anhui, Guangde, Shanbeixiang, Jishan Cun, Nianzhiwu		31.0675 119.4432	[20]
Sichuan: Anning River Valley (China)—SAV						
KF279407	ADL		Liangshan (near Qionghai sea) Yanjia Buzi (Heyan Road)		27.87505 102.30867	unpub
Sichuan Plain (China)—SCB						
HM120846	FCC		Meishan, Dongpo, Baiyang Cun		30.0373 103.9002	[20]
Taiwan—TAI						
KF279410			Taiwan (W) Changhua, Puyan Township		23.996416 120.475913	unpub
Yunnan: Erhai Basin—YEB						
HM120847-8; KF279408-9	FCC		Yunnan, Xizhou Chaoyang Cun; Yunnan, Xizhou, Sili Cun		25.82887 100.117770	[20]; unpub
Japan—JAP						
JQ781215		ADL	Honshu, Yamanashi, Nirasaki		35.715 138.434	unpub
Philippines—PHL						
JQ781211-12		ADL	Mindoro, Mansalay		12.547 121.442	unpub
Outgroup: *Schistosoma mekongi*						
NC002529	ADL		Lao PDR, Khong District, Ban Hat-Xai-Kuhn (Mekong River)		14.12056 105.865860	[21]

Loci sampled are *cox*1 and *cox*3 (extracted from longer contigs, including complete mitochondrial genomes). Data are: GenBank accession numbers (GACs), collection localities (with coordinates) and source references; unpub, indicates unpublished data with locations drawn from isolate codes, specimen voucher numbers or details in the GenBank. ADL indicates adult worms from laboratory lines based on field-collected infected snails, FCC stands for cercariae from snails collected in the field and used directly to infect laboratory hosts.

After submission of this manuscript, new data for Philippine and Japanese *Schistosoma japonicum* were added to the GenBank by unpublished authors of another laboratory. These data are included in Table 3, but were not included in the hypothesis testing; nevertheless, a phylogeny was estimated using this extended data set in order to assess the impact of the new data on the conclusions of this study (if any).

### Initial handling of data and selection of partitioning scheme and substitution models

Sequence data were extracted from GenBank using Biopython 1.61 Bio.SeqIO[44]. The sequences were aligned using MUSCLE 3.8.31[45], with default settings. To reduce computing time in subsequent analyses, the alignments were inspected in SeaView 4.4.2[46] and the first 240 and last 60 bps of the complete *cox*1 gene for the *S*. *japonicum* (worm) alignment were excluded (the ingroup showed no variation in these regions). Taxa with identical sequences at a locus (gene) were then excluded, leaving one representative of each haplotype: for the worms identical sequences only occurred within villages or townships, but for the snails identical sequences were found up to county level. In data sets where sequences for two loci were concatenated, data were only excluded if sequences were identical between two taxa at both loci. The final data set for the worms comprised a concatenated *cox*1+*cox*3 sequence, and had 16 taxa (19 in the extended data set) and 1994 characters (after removal of 2 identical sequences). As *cox*1 and *rrn*L data were not both available for all snail taxa, there were two data sets for the snails. The *cox*1 data set comprised 146 taxa and 599 characters (after removal of 129 identical sequences) and a second data set was made using pyfasta 0.5.2 to select all *cox*1 sequences for which there was a corresponding *rrn*L sequence; these sequences were then concatenated to produce a *cox*1+*rrn*L “both loci” alignment of 51 taxa and 1110 characters. The reading frame of the protein coding loci was determined using ExPASy Translate[47].

In addition to the alignments for individual sequences, population level data sets were produced for the snails because these were expected to be easier to visualise and detect dispersal tracts. To achieve this, the geographical range of the samples was divided into 10 biogeographical regions (see Fig 1) such that within each region there was no barrier to dispersal of *Oncomelania*; thus there would be only isolation by distance and no major ecological (e.g., lack of suitable habitat) or physical (e.g., mountain ranges or ridges) barriers; however it should be noted that the Tai Hu Plain (THP) region is likely to be interrupted by industrialised belts where there are no *Oncomelania* habitats. In contrast, each region is separated by mountains or similar areas of highland or sea. Consensus sequences were produced for the individuals within each region and a population sequence alignment estimated. The population data set included 13 ingroup taxa (JAP has two island populations, and the Philippines (PHL) was also included).

Phylogenetic analysis was performed using two fundamentally different approaches; the non-parametric Maximum Parsimony (MP) approach and the parametric Maximum Likelihood approach. Two contrasting methods were used so that resilience of the hypothesis to changes in method and associated assumptions could be used to reveal weakly supported clades. The rationale was that any clade that was represented in phylogenies found by both methods (and well supported) would be a reliable reconstruction of phylogenetic history for these taxa. In addition, the strategy helps reveal artefacts associated with a specific class of methods.

### Phylogenetic analysis by Maximum Likelihood (ML) with RAxML

RAxML 7.4.8[48] was chosen to implement the ML analysis because, among currently available programs, RAxML has shown best performance in terms of inference times and final likelihood values, and has a rapid boot-strapping algorithm which allows clade support to be estimated in reasonable time frames, even when estimating null distributions. A series of test runs were used to determine optimum values or states for the settings of the RAxML analyses (details published elsewhere[30]). The apparently optimum partitioning strategy and evolutionary model for each partition was determined using PartitionFinder 1.0.1[49], under a BIC criterion and models restricted to those implementable in RaxML. The chosen models for the snails were: *cox*1, GTR+G codons CP_123_; *cox*1+*rrn*L, GTR+G codons CP_123_ and *rrn*L separately partitioned; populations, GTR+G codons CP_111_ *cox*1 and *rrn*L partitioned separately. For the worms the models were, for *cox*1+*cox*3, GTR+G codons CP_112_ with *cox*1 and *cox*3 codons in the same partition (i.e., there were two only partitions). RAxML was run with 100000 bootstrap replicates, using every fifth tree found by bootstrapping as a starting tree for a series of 20,000 full ML searches. The CAT approximation in RAxML was not used. Three main runs were performed with different random number seeds. After checking that the independent runs led to trees of the same topology and very similar levels of support (±1%), the bootstrap trees for all three runs were pooled and a 100% (strict) majority rule consensus tree reported.

For the hypothesis testing, a data set was constructed that included all snail taxa for which there was a worm sample taken at the same locality. These data included ten taxa and 1994 characters. The model and partition scheme for the worms was the same as for the full worm data set, and for the snails a two partition model was again chosen: GTR+G (*rrn*L, c*ox*1codon1, *cox*1codon2) and *cox*1codon3.

### Phylogenetic analysis by Maximum Parsimony (MP) with POY

POY 5.1.1[50] (parallelised build) was used to implement a Maximum Parsimony approach. The use of MP afforded an analysis free of the assumptions underlying ML methods, and the use of POY with its dynamic homology approach (where characters (transformation series) are inferred during the process of phylogenetic reconstruction) frees the analysis of any effects particular to the alignment inferred by MUSCLE[51]. A sensitivity analysis was used to choose the weighting (gap cost etc) and partition schemes for each data set, protocol published elsewhere[52].

### Hypothesis testing

In order to test whether the evolution of the DNA sequences was consistent with a particular hypothesis, such as coevolution of *Oncomelania hupensis* and *Schistosoma japonicum*, the Incongruence Length Difference test[53] (ILD) was used as implemented in PAUP* 4.0b10[54], with 5000 replicates. The test employed a sub-set of the data which included only polymorphic sites (for reasons published elsewhere[55]). The test, which randomly exchanges segments between the snail and worm data partitions, should give ML outcomes which are not significantly different from those of the original data if the snails and worms evolved to a common history. The ILD has been shown to be rather conservative when used as a test of topological congruence if phylogenies with trees of similar topologies are compared, with the opposite effect observed if the trees compared differ markedly in structure (e.g., internal branch length differences)[56,57]. Noise in the phylogenetic signal can also lead to type-I errors in the ILD test[58]. Consequently, the hypotheses were also tested using Monte Carlo simulation in the manner of the SOWH test[59]. In the case of the test for coevolution, the test statistic is the likelihood ratio of the phylogeny inferred for the worm data (unconstrained) and the same phylogeny inferred with the evolutionary history constrained to that of the snails (represented by the ML tree estimated for the snail data). A null distribution of the test statistic was calculated by simulating many data sets using Seq-Gen 1.3.3–1[60] and the ML parameters of the substitution model inferred for the worm data, but constrained under the null hypothesis (the ML tree for the original worm data constrained by the snail ML tree). For each simulacrum the ML tree was estimated both unconstrained and constrained by the snail ML tree, and a likelihood ratio computed. The null distribution then being a distribution for the amount of discord expected to occur when the worm phylogeny had evolved according to the same history as the snails. If the likelihood ratio for the original data falls above the 95^th^ percentile of the null distribution, the hypothesis that both worms and snails evolved to the same (i.e., the worms') phylogenetic history can be rejected at *P*<0.05. The replicates were performed using RAxML (with settings as for the original worm data, but bootstrapping set to terminate according to a convergence criterion based on the extended majority rule consensus trees), and they continued until the null distribution appeared to have stabilised, as judged by a plateau of *t*-values with increasing replicate number.

### Visualisation of phylogenies and phylogeographies

In cases where the topologies resulting from phylogenetic analysis by ML and MP did not agree for a particular data set, a strict consensus tree was generated from the two trees so that discordant clades were represented by polytomies. Consequently, the resolved clades in the final trees for each data set were those supported by both methods. In order to visualise the phylogeographies of both snails and worms, the phylogenies were mapped in Marble Virtual Globe 1.8.3 using the Phylo2GE R script. In addition, phylogenies were compared (topologically) using the compare2Trees algorithm[61], which scores each pair of branches, between the trees, according to the common taxon partitions they define, with the branches then paired so as to maximise the overall score; this process yields a score (S) value for a pair of trees.

## Results

### Phylogenetic reconstructions

To enable RAxML and reduce computing time 129 identical and/or ambiguous *cox*1 sequences were excluded from the original snail data set (i.e., the sequence alignment for *Oncomelania*) to leave 146 taxa. Only one of the 129 exclusions involved identical sequences at the inter-regional scale. The haplotype diversity by region was roughly inversely proportional to the sampling intensity (i.e., number of sequences per region), except for YEB and PHL, and to a lesser degree SCB, which had small sample sizes and low haplotype diversities (Table 4).

**Table 4:** Numbers of individuals sequenced (N) and haplotype diversity (H) by region for the *Cox*1 and *rrn*L data sets (snails) and the *Cox*1+*Cox*3 data set (worms).

	*Cox*1		*rrn*L		*Cox*1+*Cox*3
Region	N	H	N	H	N
FCP	2	1.00	2	1.00	0
GCP	5	0.80	1	1.00	0
DLB	74	0.55	5	0.80	2
PLB	12	0.92	3	1.00	1
THP	69	0.45	3	1.00	5
SAV	43	0.47	32	0.84	1
SCB	20	0.50	12	0.75	1
TAI	14	0.71	1	1.00	1
YEB	15	0.47	4	0.50	4
JAP	7	0.86	5	1.00	0
PHL	4	0.50	2	1.00	0

The haplotype diversity for the worms was 1.00 for all regions. Region codes are described in Fig 1.

Replicate phylogenetic analyses run for the snail data, with different random seeds and only the *cox*1 data, failed to result in a common tree (S<0.88), and there was poor agreement between the results of the ML and MP searches (S<0.55); the phylogeny also contained many unresolved clades. Consequently, the *cox*1 data were considered to lack phylogenetic signal and were not considered further in this study. In contrast, the phylogenies estimated for both loci (*cox*1 and *rrn*L concatenated) showed good agreement among replicate runs (S>0.92) and between ML and MP (S>0.75).

**Fig 2:**
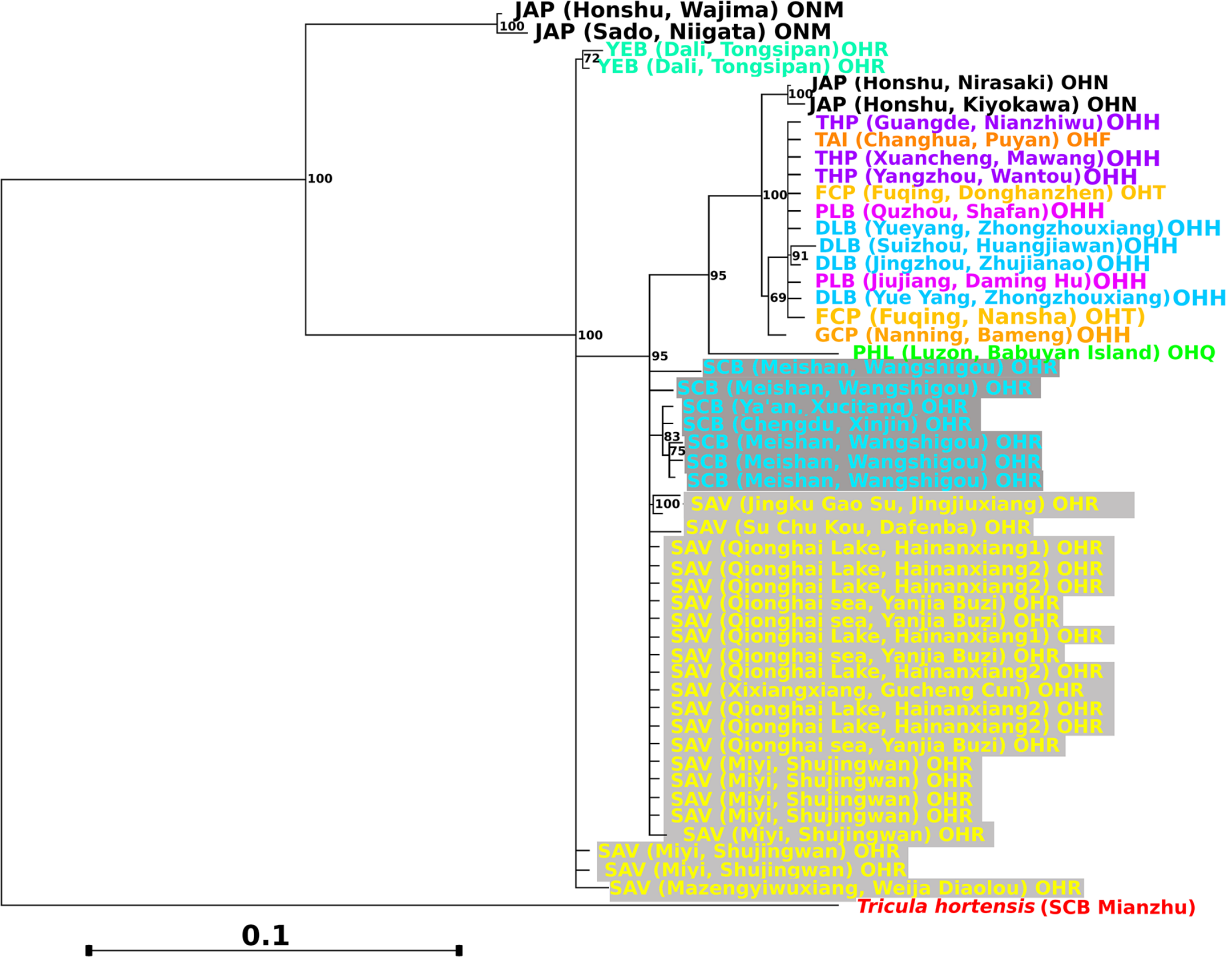
Phylogram representing the strict (100%) consensus tree between Maximum Parsimony (MP) and Maximum Likelihood (ML) phylogenies. As estimated for the *Oncomelania* populations sampled at both loci, *cox*1 and *rrn*L, with clade support values averaged from 100000 bootstrap replicates (ML) and 5000 jackknife iterations (MP). The colours assigned to the taxon names correspond to those of the biogeographical areas mapped in Fig 1. The light and dark grey shading denotes the western mountain clades. The outgroup is in red.

**Fig 3:**
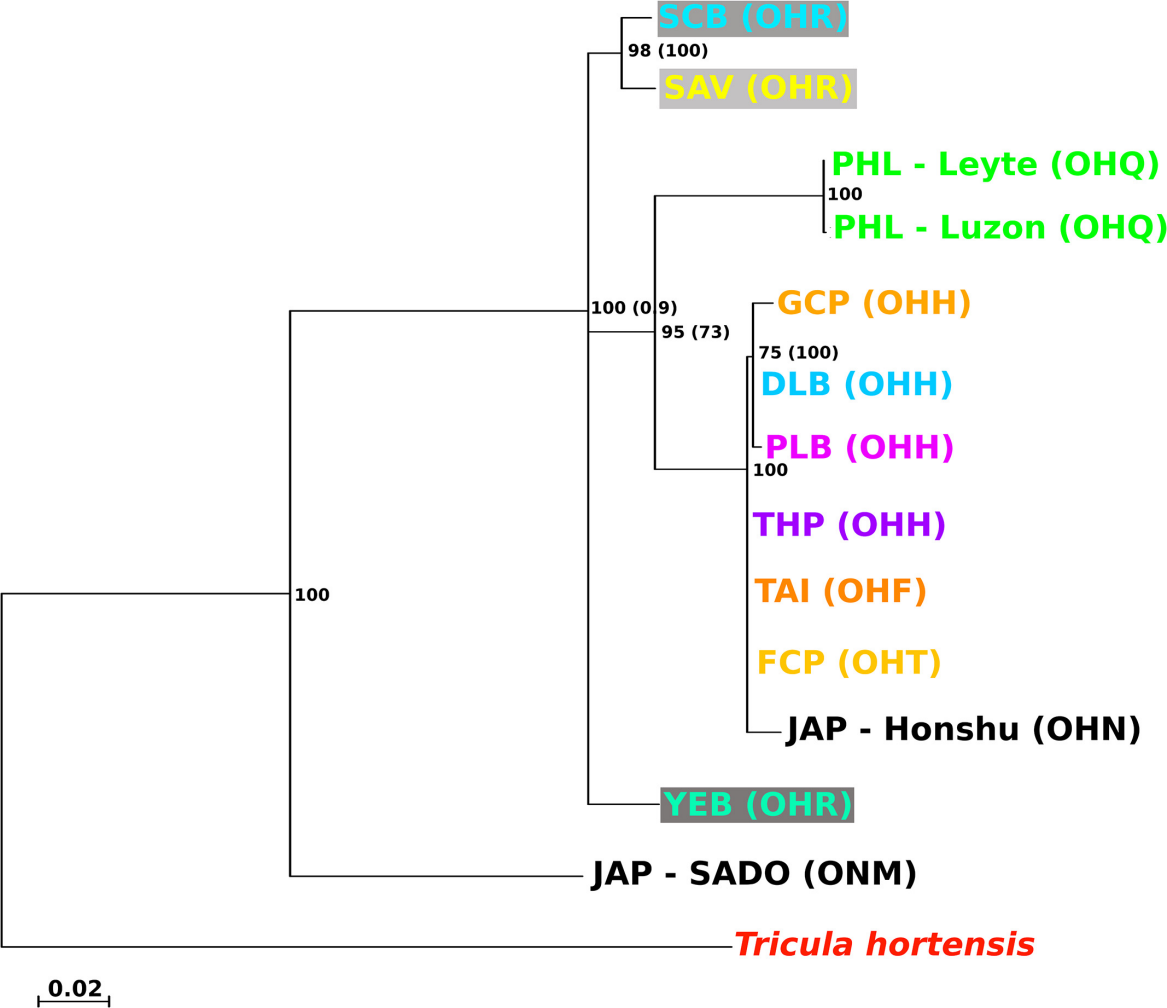
Population phylogeny for *Oncomelania* at both loci *cox*1 and *rrn*L. Conditions of the analyses and colour scheme follow Fig 2, except that the DNA sequence data for all individuals were pooled into a consensus sequence for each biogeographical region before the sequences for the two loci were concatenated.

The strict (100%) consensus tree between the ML and MP searches is given in Fig 2. Considering *O*. *hupensis*, the only biogeographical regions characterised as monophyletic clades are JAP and YEB. Nevertheless, the Sichuan populations (SCB and SAV), which lie in an isolated mountainous area, form an unresolved near-monophyletic clade, except for three SAV individuals which form a further unresolved clade at the base of the *O*. *hupensis* clade (this could be a result of long branch attraction due to saturation or a lack of apomorphies in younger clades[62], with slippage of long branches leading to SAV taxa, towards the root of the tree). The YEB populations, also mountainous but separated from the Sichuan populations by the Hengduan Range, formed a distinct basal clade at the same level as the three extraneous SAV samples. Thus, the basal clades of the phylogeny are occupied solely by *O*. *h*. *robertsoni* (and *O*. *minima*). The remaining major clade, which appears derived from *O*. *h*. *robertsoni* contains all the other *O*. *hupensis* samples, including the non-Chinese taxa *O*. *h*. *quadrasi* (Philippines), which is basal to the clade, and *O*. *h*. *nosophora* (Japan)–suggesting that these taxa did not diverge *in situ*. Although *O*. *h*. *nosophora* is monophyletic and *O*. *h*. *robertsoni* is near monophyletic (it's apparent polyphyly might be explained by long branch attraction), *O*. *h*. *hupensis* is polyphyletic and includes *O*. *h*. *tangi* and *O*. *h*. *formosana* (of FCP and Taiwan, respectively); this suggests that the latter two may be populations of *O*. *h*. *hupensis*. The GCP taxon lies at the base of the Chinese *O*. *h*. *hupensis* clade and this gives some support to the case for *O*. *h*. *guangxiensis*. The populations of the lake and marshland region (DLB, FCP and THP) form an unresolved clade, suggesting that there are few barriers to migration (gene-flow) between them. In view of this, the population phylogeny (where data for individuals is pooled to provide consensus sequences for each region) provides a representative summary of the phylogeny (Fig 3). The population phylogeny agrees with that for both loci, based on individuals, except that it shows GCP as forming a sub-clade, of the Lake and Marshland, Taiwan and Japan clade, with PLB and DLB.

**Fig 4:**
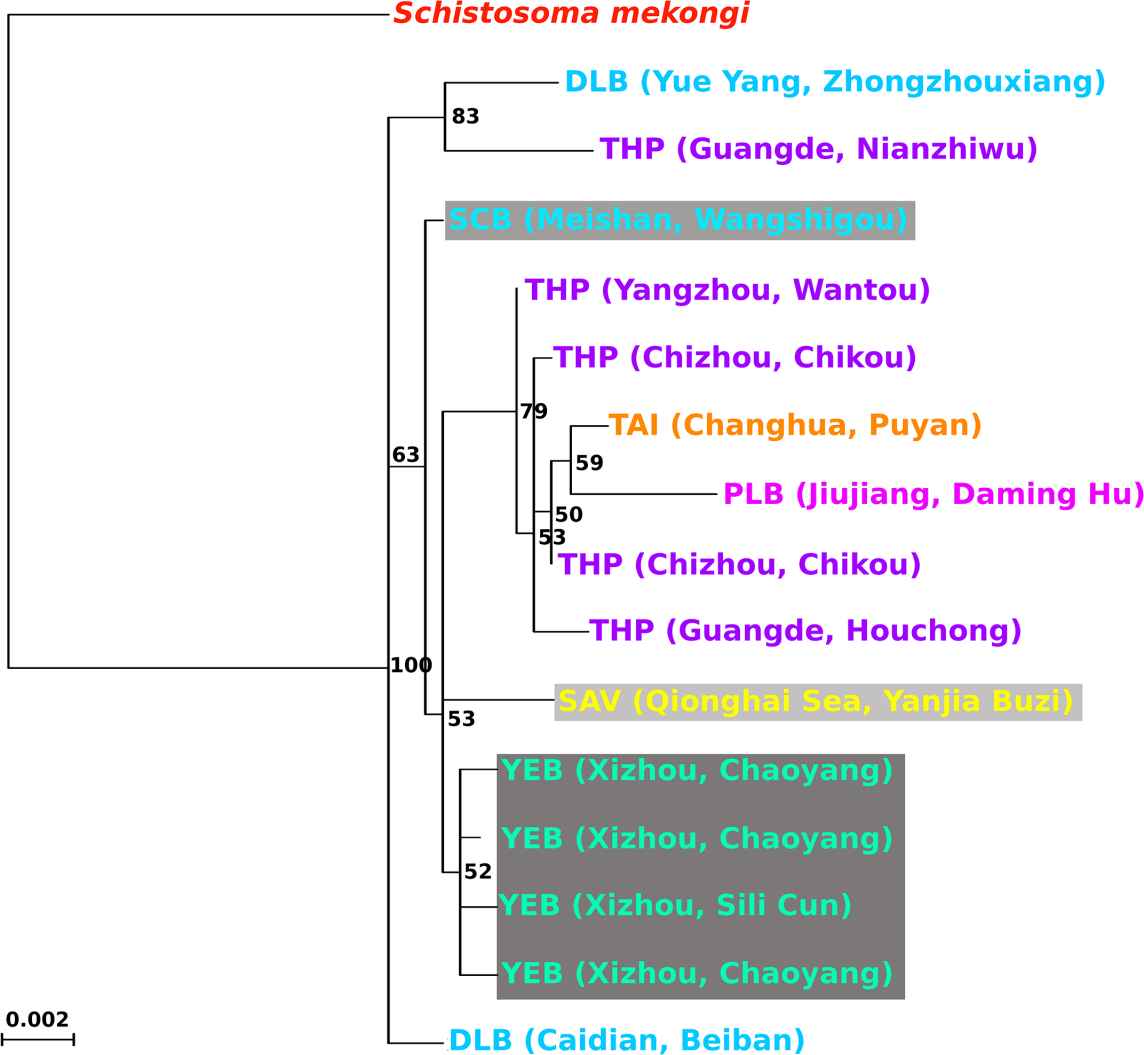
Phylogeny estimated for *Schistosoma japonicum*. Conditions of the analyses and colour scheme follow Fig 2.

The phylogeny estimated for the worms (Fig 4) differed from that of the snails in certain key features. For example, some DLB and THP populations are basal to the western mountain clades (YEB, SCB and SAV). As in the snail phylogeny, the Taiwan population falls within a clade comprised solely of Lake and Marshland mainland Chinese taxa; however, this clade excludes DLB and so contains only THP, PLB and TAI, also the Taiwan sample clusters with PLB forming a clade derived from the Chikou THP samples.

### Hypothesis testing

**Fig 5:**
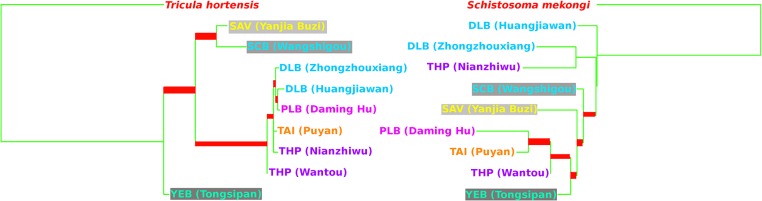
A comparison of the snail (left) and worm (right) population phylogenies. Clades which do not occur on both trees are marked in red (based on the output of compare2Trees). Conditions of the phylogenetic analyses and colour scheme follow Fig 2.

The snail and corresponding worm phylogeny (i.e., that estimated in exclusion of taxa not held in common; Fig 5) showed a low level of correspondence (S = 0.46). Consequently, it was necessary to test the null hypothesis that the snails and worms had evolved to a common evolutionary history, i.e., that of the snails. Initially the ILD test was used to detect significant conflict in the phylogenetic signals of the snail and worm data. The test was significant (*P* = 0.00004) suggesting that the two data partitions are the result of different evolutionary processes. To test further the null hypothesis, a SOWH test was performed; this resulted in a likelihood ratio for the observed data (unconstrained / constrained by the null hypothesis tree) of 75.52 and a 95^th^ percentile for the null distribution of 4.26. Consequently, the null hypothesis can be rejected in the light of these data (*P*<0.0001). In view of these findings it appears highly unlikely that the evolutionary radiation of *Schistosoma japonicum* across China was shaped or driven by that of the snail intermediate hosts.

### Phylogeography

**Fig 6:**
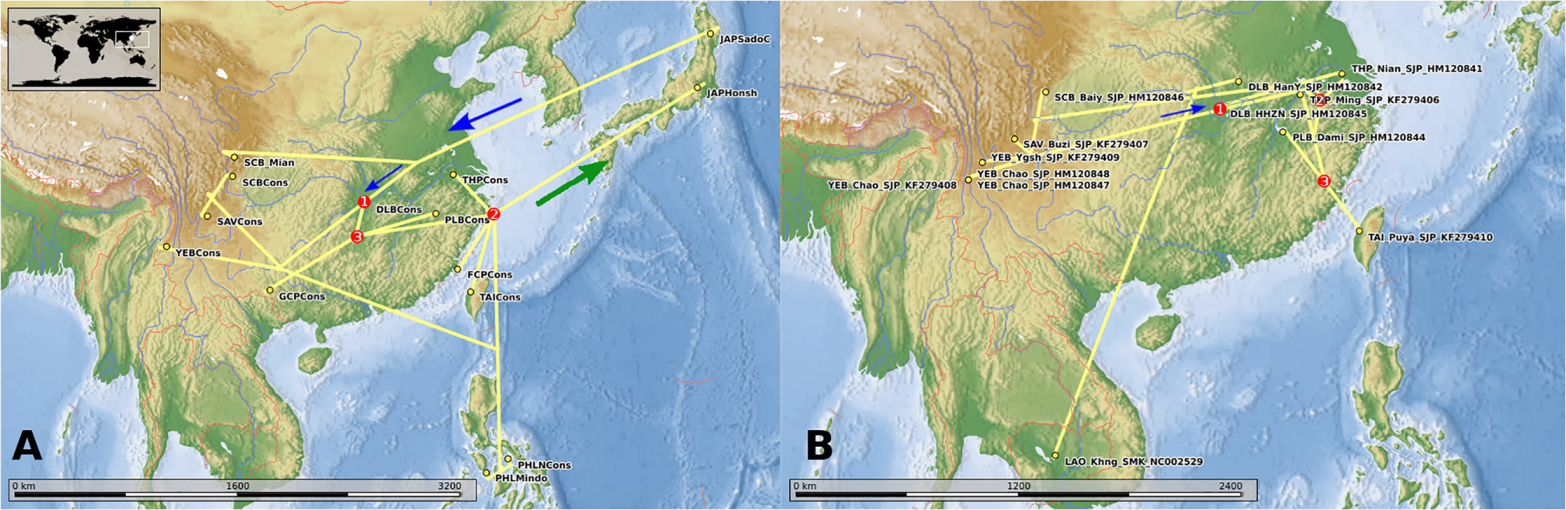
Projection of the phylogeny for the *Oncomelania* (A) and *Schistosoma japonicum* (B) populations onto a topographic map. Plotted using Marble Virtual Globe (open source). The nodes in red indicate key divergence events and are numbered chronologically (see Discussion for details). The blue arrows show the polarity of initial radiation according to the East to West hypothesis and the green arrow a later back colonisation. For an interactive projection please use the kml files for these maps (S1 Dataset) provided in the Supporting Information.

Geospatially projected phylogenies (Fig 6 and Files A and B in S1 Dataset) assist in phylogeographical interpretation and in the present study they reveal clear differences between the radiations of the snails and the worms. The map for the snails (Fig 6A) suggests initial colonisation of the valleys of the western mountains (SAV, SCB, YEB) by a proto-*Oncomelania hupensis robertsoni*; thereafter these lineages, established in their respective valleys and basins, appear to have stabilised after initial divergence (no further cladogenesis), and remained so throughout most of the history of *O*. *hupensis*. The mountain clade appears to have given rise to the Lake and Marshland and East-coast clades (THP, FCP) of Chinese *O*. *h*. *hupensis*, with radiations back into Japan (as *O*. *h*. *nosophora*) and to Taiwan and the Philippines more recently in the history of this species. A second, slightly more recent, radiation from DLB, west to GCP and eastwards to PLB, then occurs. In contrast the worms show a history where the Lake and Marshland (eastern) populations appear to involve cladogenic events that occur throughout the history of Chinese *S*. *japonicum*, whereas the western mountain taxa are stable following initial establishment in their respective valleys. Unlike the snails, the worms show a most recent colonisation event of Taiwan that is associated with the PLB region rather than THP/FCP.

After submission of this manuscript, new data for Philippine and Japanese *Schistosoma japonicum* were added to the GenBank. In order to determine the position of these additional populations in the phylogeography and their congruence with the snail phylogenies, an extended data set was subjected to phylogenetic analysis. File C in S1 Dataset and S1 Fig give the resulting kml and an image showing the extended phylogeny projected onto a globe, respectively.

## Discussion

### Taxonomy and phylogenetics

Of the four Chinese *Oncomelania hupensis* sub-species described by Davis[9], only two are supported in the present study. *Oncomelania h*. *robertsoni* is not polyphyletic and all individuals sampled of this sub-species are basal in the phylogeny; therefore, this taxon is supported. In contrast, *O*. *h*. *hupensis* is polyphyletic and includes *O*. *h*. *tangi* and *O*. *h*. *formosana*; this suggests that *O*. *h*. *tangi* and *O*. *h*. *formosana* are not valid sub-species, but are *O*. *h*. *hupensis*. Indeed *O*. *hupensis* populations can demonstrate considerable differences in morphology (mostly of the shell) and yet cluster together genetically[11]; therefore it is possible that all three sub-species in the large derived clade indicated by phylogenetic analyses for both loci and for snail populations (Figs 2 and 3) are in fact *O*. *h*. *hupensis*. The phylogeny estimated for the worms, in which the lowland populations form a clade distinct from those of the highland populations (SAV, SCB and YEB), is consistent with morphological, host-utilisation, and maturation rate observations that suggested independent lineages or sub-species for the highland and lowland *S*. *japonicum* in China[63]. Nevertheless, *O*. *h*. *hupensis* appears paraphyletic with two DLB (and one THP) populations forming separate clades near the base of the tree. Divergence of some Hunan/Hubei (DLB) populations away from those of other Lake and Marshland regions could result from the particularly long history of intensive control efforts[4] in these provinces, with slippage of these clades towards the outgroup owing to long branch attraction coupled with a lack of apomorphies among younger clades[64]. It is also interesting to note that the zoophilic strain[65] from Taiwan is not genetically distinct from those strains capable of infecting humans, although it is one of the two most derived members of the lake and marshland clade.

### Hypothesis testing

The lack of concordance between the snail and worm phylogenies found in the present study could result from heterochronous evolution of the host and parasite in response to different palaeo-geographical or climatic environments. In the closely related taxon, *Schistosoma mekongi*, the radiation of the parasite (dated 2.1–1.0 Ma) was shown to be independent of that of its intermediate host (10–5 Ma). Divergence events among the snails were considered to be a concerted response to the final Indosinian orogeny around 5 Ma, with *S*. *mekongi* colonising the snails across its present range much later (early Pleistocene)[31,32]. Using a molecular clock the introduction of *O*. *hupensis* across mainland China has been dated to the early Miocene (*c*.*a*., 22 Ma), with high rates of cladogenesis 8–2 Ma and linked to the exceptionally warm and humid climate in the region at that time and tectonic upheaval in Japan[30]. The divergence of the *Schistosoma japonicum* clade has been dated at 4.6 Ma[32]; this, implies that the radiation of *O*. *hupensis* occurred before that of *S*. *japonicum*. If the radiation of the snails and worms is heterochronous there is no opportunity for coevolution; the implication is also that ancestral intermediate hosts differed from those of the present, which again makes coevolution unlikely.

Coevolution might be expected in *Schistosoma* species such as *S*. *mansoni*, which infect snail populations at relatively high prevalence and achieve high rates of cercariogenesis[66], but seems unlikely in *S*. *japonicum* because of its lower prevalence in the intermediate host populations. The prevalence of natural infections in China ranges from 0.038% (Jiangsu in 2011) to 7.8% (Anhui in 2013)[67]. In cases where the snails experience a low probability of becoming infected, they are under little pressure to invest resources in defence[68]. In contrast, prevalences as high as 75.7% have been reported for natural populations of *S*. *mansoni* in *Biomphalaria glabrata* in Brazil[69]. Factors such as the generalised nature of gastropod immune systems, and evidence for frequent host switches in the parasites’ evolutionary histories, also make a “Red Queen” scenario unlikely for these schistosomes[32,68,70,71]. Consequently, molecular clock dating for other members of the *S*. *sinensium* clade and their intermediate hosts (also close relatives of *Oncomelania*) and the low prevalence of infection in *O*. *hupensis* suggest that the lack of evidence for coevolution found in this study is to be expected.

It could be argued that comparison of naturally infected (and infective) snails, rather than snails from the local populations transmitting the parasite (but not necessarily themselves infected) would be more likely to reveal signs of coevolution, i.e., there could be sub-populations or sub-strains of snails that have been coevolving with the worms, rather than the general snail populations sampled in this study. The existence of such sub-populations is questionable, as is the existence of resistant and susceptible lineages of snails in schistosomiasis. It has been shown that any snail taken from a natural population of *B*. *glabrata* will become infected if exposed to enough miracidia from a natural *S*. *mansoni* population[72]. Consequently, a “resistant” snail line is merely a sub-population selected to be discordant with the epitopes expressed by a particular *Schistosoma* line. Even authors working on the *B*. *glabrata*-*S*. *mansoni* association, have observed a complete reversal in resistance phenotype after a few laboratory generations and note that genotypic responses would only occur in associations where prevalences are high[36]. In view of this, it is unlikely that resistant sub-strains of *O*. *hupensis* exist and infection probably occurs more at random across the general snail population (perhaps influenced by ecology and spatial coincidence). Nevertheless, it would be interesting to repeat the present study using naturally infected snails from the localities studied and seek to detect any signs of coevolution.

### Phylogeography

As mentioned above, earlier studies date the introduction of *O*. *hupensis* to mainland China at around 22 Ma, at which time the region was significantly less mountainous, and three general clades appear to have been rapidly established; these span China, with the *O*. *h*. *robertsoni* clade in the far West, the Dongting Lake Basin (DLB) clade in the center of the Lake and Marshland region, and the Tai Hu Plain (THP) clade near the East coast of mainland China (Fig 6A). *O*. *h*. *robertsoni* appears to have diverged little since its initial colonisation of the mountain valleys in which it is found today, and its lineages have probably been isolated therein since the second major uplift of the Himalaya about 7 Ma[73]. The other two clades appear to have undergone two successive, more recent, cladogenic events, which in the case of the DLB clade, gave rise to the GCP and PLB populations (to the West and East, respectively; Fig 6A node 3). The THP clade gave rise to FCP, Taiwan, Japanese and Philippine populations of *O*. *h*. *hupensis* (Fig 6A node 2). Such a recolonisation of Japan by mainland Chinese *O*. *hupensis* is consistent with the “East to West” hypothesis[29]. The divergence of the western clades is likely to have occurred around 8 Ma when the Himalayan uplift altered global climate and triggered increasing aridity in the region, which would have fragmented existing *Oncomelania* populations[30]. Interestingly, the Taiwan population is also included in the lake and marshland clades even though Taiwan has been separated from the mainland since Pleistocene[74,75]and the more recent divergences within *O*. *hupensis* occurred before 2 Ma. It is possible that tsunami events could have exchanged snails between the mainland and Taiwan. Indeed, the March 2011 Pacific tsunami demonstrated that large aggregates of material may cross even oceanic distances in less than 15 months and that freshwater pools on these may harbour viable communities of exotic aquatic organisms (including molluscs)[76]. *Oncomelania* is also capable of aestivating out of water for several months. In addition, intermittent land bridges occurred, linking Taiwan during the Quaternary[77]. The relative lack of genetic variation among the Taiwan populations also suggests a recent colonisation of the island (or extinction of long established lineages followed by recent recolonisation). The Guangxi Plain (GCP) populations formed a clade distinct from other *O*. *hupensis* and may have been isolated from the other *O*. *hupensis* taxa since the late Miocene/late Pliocene by uplift along the margins of the Youjiang Basin (Jiangnan range)[78,79].

Although the radiation of *S*. *japonicum* is described above as occurring around 4 Ma later than that of the snails, the western (mountain) clades of the parasite still show the same initial divergence and then absence of cladogenesis as do the snails. The ancestral hosts of *S*. *sinensium* group parasites appear to be rodents (especially *Rattus* and its sister group) and it is therefore likely that *S*. *japonicum* radiated in China in concert with the Pliocene radiation of *Rattus* which began in Southeast Asia[80]. After colonising the valleys of Sichuan and Yunnan in rodents radiating into China from Southeast Asia, the parasites would become isolated in these valleys by cooling and increasing aridity in the Pleistocene[81]; thus suppressing further cladogenesis. In contrast, the lake and marshland clades undergo a series of cladogenic events spread from around the early Pliocene towards the Recent. Initially DLB and THP clades are established, together with a second THP clade that is derived from the *O*. *h*. *robertsoni* clades (Fig 6B node 1), this is followed by progressive diversification of one branch of the second THP clade (Fig 6B node 2), whilst the DLB-associated THP clade remains stable. The second THP clade most recently gives rise to an ancestral form that diversifies into a PLB and a Taiwan clade (Fig 6B node 3). The possibility of a long-distance dispersal from Sichuan in the western mountains to THP near the East coast of China (Fig 6B arrow) is an intriguing one; however, the possibility of misidentification or laboratory error concerning Genbank deposited sequences must also be considered where highly inconsistent relationships are found The introduction needs to be dated in future work and might be related to traffic down the Yangtze river (human activities) and the extensive cladogenesis and spread of the Sichuan strain in the coastal lowland areas is an unexpected event, which might relate to better development of the parasite in naïve human hosts (or presence of a more dynamic host population than in the mountain regions).

Analysis of the extended data set (S1 Fig) shows the Japanese *S*. *japonicum* arising from the same basal THP lineage as the Taiwan population. In contrast the Philippine clade arises from the basal YEB clade along with taxa from Sichuan. Although this relationship appears to mirror that of the snail phylogeny, it is a relatively recent divergence in the parasites and a relatively earlier one in the snails; thus it does not increase the degree of phylogenetic congruence between the hosts and parasites.

The possibility of extensive radiation and dispersal, after long-distance introduction of a Sichuan strain of *S*. *japonicum* to the coastal region, is important in view of the fact that the mountains of Yunnan and Sichuan appear to have formed a barrier to dispersal of *O*. *h*. *robertsoni* transmitted *S*. *japonicum* for perhaps millions of years. The observation is particularly relevant in the context of the South-to-North-Water-Transfer project (Eastern Route) which will transfer water from endemic areas in Jiangsu province to areas in Shandong Province, towards the Yellow river, where *O*. *hupensis* has yet to be found but where conditions appear to be favorable for transmission[82]. *Oncomelania* (and the associated schistosomiasis) are most widespread in the lake and marshland areas of the middle and lower Yangtze river drainage; the distribution of snail and parasite in the mountainous areas of Sichuan in more patchy, and in Yunnan they are found only in a restricted area around Dali[83]. As the GMS road projects will breach the mountain ranges between Sichuan and eastern China and Sichuan and Yunnan (and further South into Laos and Thailand), it is important to understand the colonisation history of *S*. *japonicum*.

### Conclusions

The present work has led to the rejection of the hypothesis of coevolution for *Schistosoma japonicum* and *Oncomelania hupensis* on the basis of the samples available. The finding is consistent with models regarding the relative timing of the radiations of the two groups proposed in earlier studies, and with observations of a low prevalence of infection in these snails. Nevertheless, it is still possible that the parasites show some adaptations to a snail population through which they have been cycling for some time, but the analysis does suggest that the worms are not highly evolved/restricted to a particular sub-species or strain of snail. Consequently, host-switching or acquisition is more likely than would be implied by a Red Queen scenario. The findings also suggest that *O*. *h*. *formosana* (of Taiwan) and *O*. *h*. *tangi* (of Fujian) might be *O*. *h*. *hupensis* and not distinct sub-species. The phylogeographical reconstructions suggest that at least one long-distance dispersal event occurred across China between the western mountain populations of *S*. *japonicum* and the East coast region. The event appears to have triggered extensive cladogenesis and dispersal (including to Taiwan) on the East coast. Consequently, further work is necessary to confirm and to date this long-distance dispersal and to detect any further such events, so that their origins and driving forces can be determined. Further work is also required with a richer data set as support for some of the clades indicated in the analyses was less than 90%. The findings are particularly important in view of the infrastructure development plans which will breach the mountain barriers between Sichuan, Yunnan and Southeast Asia. The results also have implications for the spread of *S*. *japonicum* as, in the absence of coevolution, the parasite may more readily colonise new snail populations to which it is not locally adapted, or even new intermediate host species, and this can facilitate its dispersal into new areas. The work also lends support to the East-West hypothesis for the origin and dispersal of *Oncomelania* and the Pomatiopsidae.

## Supporting Information

S1 Dataset An archive containing kml files to project the phylogenies onto Google Earth or any kml enabled virtual globe. File A, *Oncomelania* populations; File B, *Schistosoma* populations; File C, extended set of *Schistosoma* populations.
(ZIP)

S1 Fig Projection of the phylogeny for the extended set of *Schistosoma* populations onto a topographic map. Plotted using Marble Virtual Globe (open source). For an interactive projection please use the kml file File C in S1 Dataset also provided in the Supporting Information.
(PNG)
