# Supplementary figures and images for: Comparative Phylogenetic Studies on Schistosoma japonicum and Its Snail Intermediate Host Oncomelania hupensis: Origins, Dispersal and Coevolution
Source: PLoS Negl Trop Dis. 2015 Jul 31;9(7):e0003935. doi: 10.1371/journal.pntd.0003935 (PMC4521948; doi:10.1371/journal.pntd.0003935)

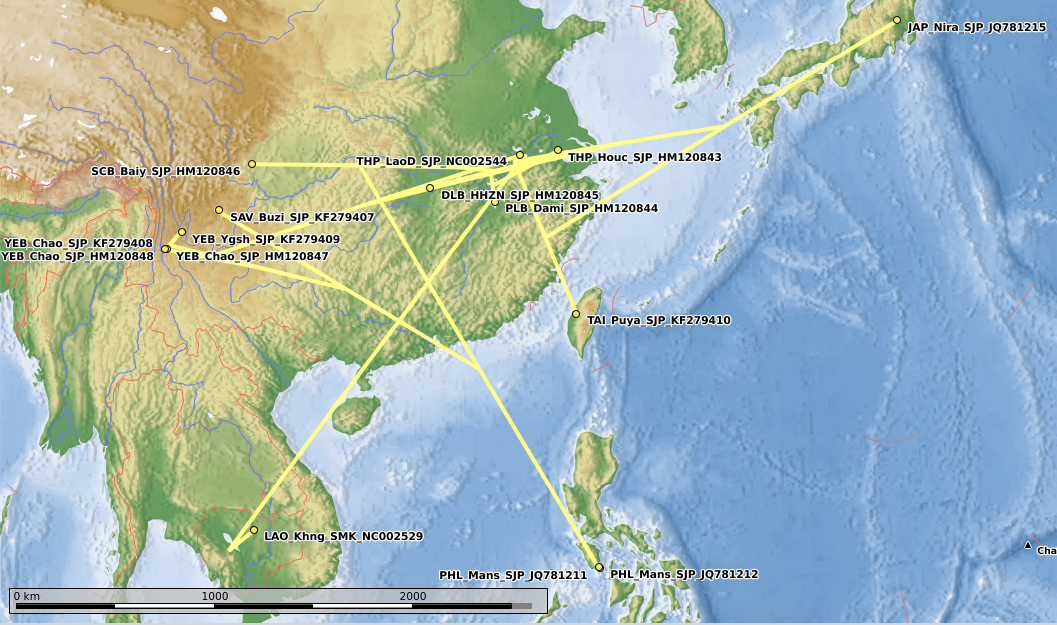

Supplement: S1 Fig — Plotted using Marble Virtual Globe (open source). For an interactive projection please use the kml file File C in S1 Dataset also provided in the Supporting Information. (PNG) [file pntd.0003935.s002.png]
